# The relation between statistical power and inference in fMRI

**DOI:** 10.1371/journal.pone.0184923

**Published:** 2017-11-20

**Authors:** Henk R. Cremers, Tor D. Wager, Tal Yarkoni

**Affiliations:** 1 Department of Clinical Psychology, University of Amsterdam, Amsterdam, Netherlands; 2 Department of Psychology and Neuroscience, University of Colorado at Boulder, Boulder, Colorado, United States of America; 3 Department of Psychology, University of Texas at Austin, Austin, Texas, United States of America; University College London, UNITED KINGDOM

## Abstract

Statistically underpowered studies can result in experimental failure even when all other experimental considerations have been addressed impeccably. In fMRI the combination of a large number of dependent variables, a relatively small number of observations (subjects), and a need to correct for multiple comparisons can decrease statistical power dramatically. This problem has been clearly addressed yet remains controversial—especially in regards to the expected effect sizes in fMRI, and especially for between-subjects effects such as group comparisons and brain-behavior correlations. We aimed to clarify the power problem by considering and contrasting two simulated scenarios of such possible brain-behavior correlations: weak diffuse effects and strong localized effects. Sampling from these scenarios shows that, particularly in the weak diffuse scenario, common sample sizes (n = 20–30) display extremely low statistical power, poorly represent the actual effects in the full sample, and show large variation on subsequent replications. Empirical data from the Human Connectome Project resembles the weak diffuse scenario much more than the localized strong scenario, which underscores the extent of the power problem for many studies. Possible solutions to the power problem include increasing the sample size, using less stringent thresholds, or focusing on a region-of-interest. However, these approaches are not always feasible and some have major drawbacks. The most prominent solutions that may help address the power problem include model-based (multivariate) prediction methods and meta-analyses with related synthesis-oriented approaches.

## 1. Introduction

A fundamental assumption underlying virtually all experimental science is that the experiments one conducts are in fact capable of testing the hypotheses of interest. So stated, this assumption may seem vacuous: why would we ever bother to conduct experiments if we did not think they would—or at least *could*—work? But there is often a sizeable gap between the perceived and the actual likelihood of an experiment providing informative results. Experiments can fail for any number of reasons: poor design, faulty equipment, biased sampling, measurement error, data miscoding or loss, and so on. Many of these causes are difficult if not impossible to anticipate and can only be addressed with the benefit of hindsight. But some are not; some causes of experimental failure *can*, to a considerable extent, be anticipated. The present article addresses one such cause: low statistical power. Low power can lead to experimental failure even if in case of an otherwise *perfect* experiment, with none of the above mentioned issues. This is a problem for any type of research, but particularly for fields like neuroimaging, where the large number of variables and relatively small number of observations frequently leads to low statistical power. This problem has received previous and renewed attention [1–10], but sample size recommendations and other prescribed solutions sometimes conflict, and the topic remains controversial.

Here we review the causes and symptoms of, and potential remedies for, low statistical power in fMRI studies. We begin with a brief power primer (Box 1) and place the problem in a historical context. We then present results from both simulated and empirical fMRI analyses that aim to help better understand and visualize the nature and extent of the power problem. Finally, we end with suggestions for means of improving statistical power in future fMRI research.

**Box 1. A mini power primer**

In the traditional null-hypothesis statistical testing (NHST) framework, Type I error is the probability of incorrectly rejecting the null hypothesis, and statistical power refers to the probability of correctly rejecting the null hypothesis. That is, it is the probability of concluding that an effect is present in the population when that effect does in fact exist [11]. The complement of power is the Type II error, or false negative rate: the probability of failing to reject the null hypothesis when it should in fact be rejected. The power of any statistical test is essentially determined by three variables: the sample size of a study (n), the detectable effect size (the strength of an association between two variables [12], for instance expressed as Cohen’s d or the Pearson’s r) and the nominal Type I error rate (α) (however, when other factors (not just subjects) such as stimuli are also modelled as random factors, power is a complex function of all the random factors, see references [13,14]). Of these three variables, the effect size is typically surrounded by the greatest uncertainty (as α and n are both under experimental control to varying degrees). How strong a correlation does one expect between, say, a personality trait and amygdala activation in response to emotional faces? Effect size estimates cannot simply be conjured out of the air, or stipulated by fiat. In practice, the estimates are usually derived from previous reports of related findings.

Unfortunately, the nature of the classical hypothesis testing process makes it difficult to obtain unbiased effect size estimates. Because researchers are more likely to report statistically significant results than non-significant results [15], any influence that increases the absolute magnitude of an effect—including random error—will tend to increase its chance of being reported. Thus, effects that achieve statistical significance in a sample will, other things being equal, tend to overestimate the magnitude of the true population effect [3,16–18]. Moreover, the severity of this problem increases as statistical power drops, because the harder it is to detect an effect, the larger the contribution of chance has to be in order for the effect to attain statistical significance. The video in supplementary file S1 Video illustrates this issue by showing a medium effect size in a large sample and the variation in effect size estimates of subsamples with different sample sizes.

Another issue with classical hypothesis testing is that in the strict sense, the null hypothesis refers to a point estimate that is *exactly* zero [19]. Especially in a dense interconnected system like the brain, it seems unlikely that any effect is exactly zero [20]. In other words, one could argue that the null hypothesis can always be rejected—and consequently, Type I error doesn’t occur, so that statistical power is always 100% ([21], and see reference [22] for a compelling empirical demonstration). And even if an effect that is *exactly* zero could theoretically exist, critics have long pointed out that mere rejection of the null is generally uninformative, because the null would always be rejected if enough data were collected [21,23]. What we thus intuitively care about is not just knowing that effects are not exactly zero, but also knowing how big those effects are. As an alternative to the classical type I/II error, the type S (sign) and M (magnitude) error system has consequently been proposed [16,24]. In the current paper we will use the standard type I/II error framework due to the widespread use, but will also highlight the relation between statistical power and effect size (mis)estimation.

Lastly, when dealing with multiple dependent variables, several definition of power can be considered. One important difference is the *average* power to correctly reject the applicable null hypotheses or the power to correctly reject *at least one* null hypothesis [25,26]. The latter is naturally much higher that the former (see figure A in the S1 Supplementary Analyses). This difference between these two definitions is particularly important in the context of research like whole-brain voxel-wise fMRI analyses, where the number of independent tests often runs into the hundreds of thousands yet the number of observations is relatively low (e.g. 15–30 subjects). A failure in these *mass univariate* analyses to correct for multiple comparisons can lead to an extremely high false positive rate, so researchers typically apply much more conservative thresholds. However statistical power also rapidly decreases with more conservative thresholds. The difference between the two types of power might also explain the prevalence of underpowered fMRI studies: one could argue that in order to publish a paper one needs only a single significant finding [27], and not necessarily to identify all “active” voxels. With respect to this publication criterion, it is thus arguably an advantage in underpowered studies to test many variables and report only significant findings—even though this runs counter to the aim of comprehensive and reproducible results, and can severely distort understanding and interpretation of one’s results.

## 2. A brief history of the power problem

The most obvious problem associated with the use of relatively stringent correction levels is that, by minimizing false positives, or Type I error, one necessarily increases the rate of false negatives, or Type II error. If we require a lot of evidence before believing an effect, we are going to miss a lot of legitimate effects that just are not quite strong enough to satisfy our stringent threshold. Conventional wisdom holds that the increase in Type II error is a necessary evil, because it is a bigger sin to say an effect is real when it’s not than to say it’s not real when it is (i.e., we are willing to incur multiple Type II errors to avoid a single Type I error). This view traces back all the way to R. A. Fisher, who suggested in 1925 that “Using this criterion (of p = .05) we should be led to follow up a false indication only once in 22 trials, even if the statistics were the only guide available. Small effects will still escape notice if the data are insufficiently numerous to bring them out, but no lowering of the standard of significance would meet this difficulty” (p. 45; [28]).

Fisher and other statisticians formalized the hypothesis-testing framework at a time when statistical computation was performed manually and datasets were relatively small; thus, inferential tests tended to be applied in a confirmatory rather than an exploratory manner. However, from the definition, it follows that if a study lacks power, its authors are unlikely to identify the effects they are looking for even if those effects do indeed exist. Cohen (1962) famously showed that the typical study published in the 1960 volume of the *Journal of Abnormal and Social Psychology* had less than 50% power to detect “medium-sized” effects [29]. In other words, the success of the average experiment might as well have been determined by a coin toss. And the finding was no fluke: twenty-seven years later Sedlmeier and, Gigerenzer analyzed studies in the 1984 volume of the *Journal of Abnormal Psychology* and found that the statistical power of the average analysis remained essentially unchanged over the intervening period [30]. Similar results have been obtained, clearly demonstrating that studies in most of the social and life sciences often have inadequate power to detect anything but large effects [31–37]. Tverksy and Kahneman provided an interesting suggestion to why this problem is so persistent: researchers tend to believe that (their) small samples are more representative of underlying population effects than they actually are, which they termed the “belief in the law of small numbers” [38].

As noted above, questions about statistical power in neuroscience research in general have recently attracted considerable attention [1,6]. Nonetheless, the severity of the problem in fMRI in particular remains controversial—and the modal sample size of studies has increased only very gradually over time [6]. The relatively lackadaisical attitude with which many researchers appear to view power may in part reflect early simulation studies suggesting that the statistical power of fMRI studies with as few as 25 subjects was adequate (i.e., higher than 80%) [2,39]. However, such studies necessarily assumed very large within-subject standardized effects. The primary interest in large effects, and consequently, the sufficiency of small sample sizes to provide reasonable statistical power, has more recently been restated [7], but also criticized [8,40]. Others have suggested that concerns about Type I error may be overblown in the face of high Type II error, and have argued for more liberal statistical thresholds [41]—a move that has led to expressions of concern from still other researchers, who view such advice as lacking in any principled grounding (e.g. reference [42]).

In sum, one may easily be confused about the severity of the power problem, its consequences and possible solutions. The answer to the question of how much an fMRI researcher ought to worry about power clearly depends on the expected effect size, and effect size distribution. Moreover, most recommendations (e.g., optimistic estimates from references [2,7] consider only the relatively high-powered case of within-subject experiments (see reference [6] for an overview of within-subject effect size estimates for several common experimental designs). By contrast, we preferentially focus here on between-subject effects, since they are typically considerably lower powered, and are especially relevant to areas like personality psychology, clinical psychology and psychiatry that usually involve group comparisons or brain-behavior correlations. Below, we aim to demonstrate with simulations and empirical analyses the nature and extent of the power problem for these types of fMRI studies.

## 3. Simulated and empirical fMRI data

### 3.1 Simulation study

Although the once-heated philosophical debate surrounding modularity has subsided in recent years [43,44], a version of the question remains salient for neuroimaging researchers: to what extent should we expect standard experimental contrasts to identify individual brain regions versus diffuse networks? Is it sensible to talk about *the* brain region supporting, say, mentalizing about other people [45,46], or should we expect mentalization to activate diffuse brain networks that collectively comprise a large portion of the brain? Is a personality trait like Extraversion or Neuroticism related to activity in just the amygdala, or is it related to a network of many brain regions? Questions of statistical power may seem somewhat removed from such architectural considerations, but they actually have direct implications for our ability to determine the large-scale structure of human brain function as they relate to the proportion of brain-behavior effects that can be detected. We developed a simulation of two types of effect distribution scenarios that reflect this dichotomy and subsequently sampled from these to illustrate the relevance of statistical power to this discussion.

#### Methods

**Fig 1:**
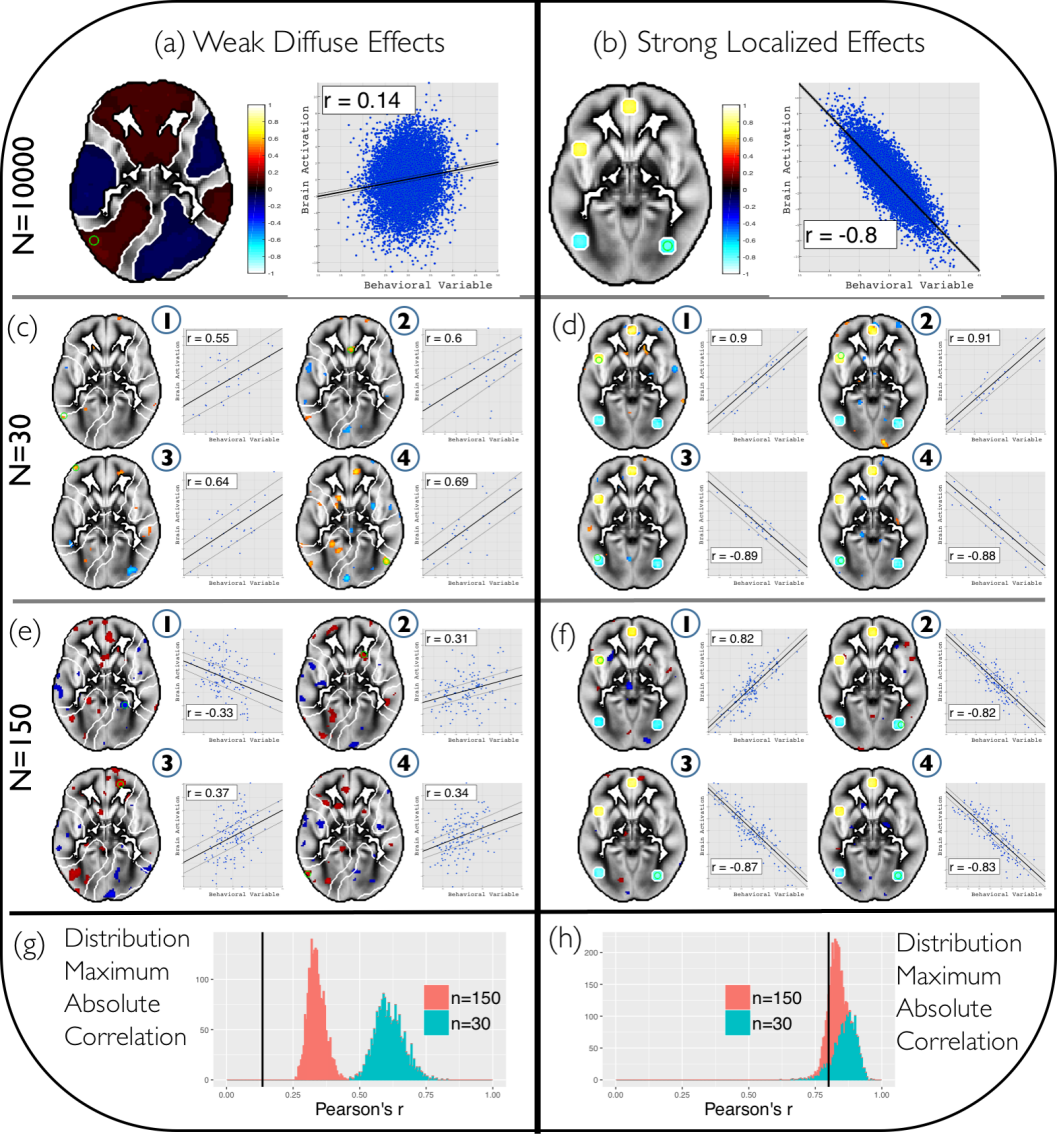
Examples of the sampling simulation. Two different brain-behavior correlation distributions (full sample n = 10.000): **(a)** Weak Diffuse (WD) and **(b)** Strong Localized (SL). The scatter plots display the voxel (indicated by a green circle in the image) with the strongest absolute correlation between its brain activity (y-axis) and the behavioral variable (x-axis). The color bar indicates the effect size (Pearson’s r). From these two full samples, 4 random subsamples are drawn with two different sample sizes: **(c)** n = 30 from the WD full sample **(d)** n = 30 from the SL full sample **(e)** n = 150 from the WD full sample **(f)** n = 150 from the SL full sample. For each subsample a *p* < .01 uncorrected threshold is applied. The distribution of the maximum effect sizes (absolute values) obtained from the subsampling procedure are shown for the **(g)** WD scenario and **(h)** SL scenario. The thick black lines indicate the maximum correlation of the full sample. Small samples (n = 30) of the WD scenario show a large discrepancy (*overestimation* of the effect sizes and *underestimation* of the spatial extend of brain-behavior correlations) with the full sample effects, and the WD subsamples appear more similar to the SL scenario. Larger samples of the WD scenario (n = 150) show less, but still substantial discrepancy with the full sample effects. For the SL scenario there is a small discrepancy between subsamples of either sample size and the full sample effects.

To investigate the relation between sample size, statistical power and several related estimates, two different simulated scenarios of effect *size* and *distribution* are evaluated. Each scenario reflects between-subjects correlations between “brain activity” and a “behavioral variable” (e.g. a personality trait) in a horizontal brain slice consisting of 4,713 voxels (see Fig 1A). First a full sample (n = 10,000) for each brain-behavior scenario is created by drawing from a multivariate normal distribution, with the specified distribution of brain-behavior covariance, spatial smoothing (a Gaussian smoothing kernel of 6 voxels FWHM was applied) and mean activation. The MATLAB code for the simulations is available on https://github.com/henkcremers/Simulate-Sampling-fMRI. In scenario 1 (*Weak Diffuse*; WD), activation in around 72% of the slice voxels shows weak correlations with the behavioral variable (range |r| = 0.05–0.14). In scenario 2 (*Strong Localized*; SL), there are strong (|r| = 0.61–0.8) positive and negative correlations in highly localized regions (3.8%) of the slice.

From these two full samples, 2,000 random subsamples are drawn with sample sizes ranging from 10 to 150. For each subsample the brain-behavior correlations are estimates applying an uncorrected statistical threshold of *p* < .01. This threshold is comparable to the commonly used *p* < .001 whole-brain analysis threshold analyses (which may involve over 200,000 voxels) and which we will apply in the HCP analyses. While the use of uncorrected thresholds remains highly controversial [42] we adopt them here purely for the sake of simplicity and to illustrate the difference between *average* and *at least one* statistical power. Obviously, correction methods to control the Family Wise Error (FWE) or False Discovery Rate (FDR) result in many fewer false positives—however, they also have correspondingly lower statistical power. In addition, a common approach is to apply cluster-extend thresholds which is in general a more sensitive method than these voxel-wise threshold methods (see for instance threshold free cluster enhancement [47]), though certain common practices have recently also been debated [48,49]. Discussion section 5.3 further briefly addresses the effects of different statistical thresholds, and in section B of the S1 Supplementary Analyses we present the results while controlling the FDR [50].

After the significance threshold of the brain-behavior correlations in the subsamples, the following estimates are derived considering the full sample brain-behavior correlations as the “true effects” (I) the *average* statistical power (fraction of true full-sample voxels attaining significance in the subsample) and *at least one* statistical power (defined as the fraction of subsamples that detected at least one true full sample effect); (II) the mean effect size (Pearson’s r) observed in voxels attaining significance, (III) the percentage of voxels attaining significance; and (IV) the average overlap of significant voxels (SV) of two subsequent samples (i,j) expressed as the dice coefficient: 2*SV^ij^_overlap_/(SV_i+_SV_j_) [51,52], which ranges from 0 to 1 and indicates the fraction of intersecting significant voxels of two images. In the current use it thus quantifies how much the results, in terms of the localization of brain-behavior correlations, of a “replication study” study overlap with the “previous” study.

#### Results and discussion

**Fig 2:**
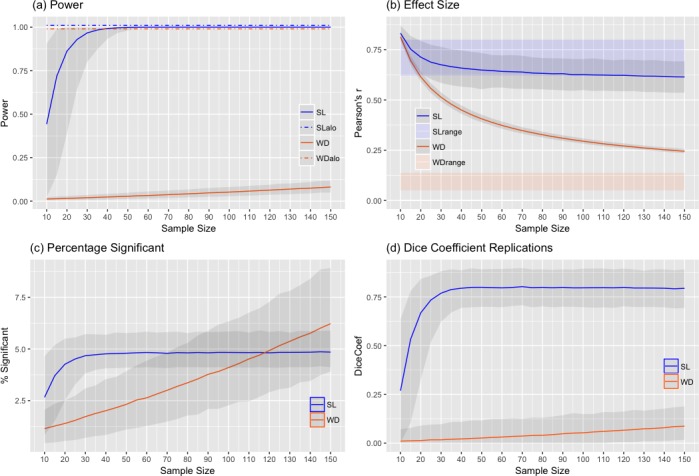
Results of the sampling simulations. Relation between sample size (n) and **(a)** *average* (solid line) and *at least one* (alo; dashed line) statistical power; **(b)** detected effect size (Pearson’s r) in the samples, and the full population effect size range (shown as a colored transparent bar); **(c)** Percentage of voxels below the threshold (*p* < .01); **(d)** mean dice coefficient: spatial overlap of significant (*p* < .01) voxels between two subsequent replications. SL; Strong Localized effects, WD; Weak Diffuse effects. The shaded grey area around the estimates reflects the 95% confidence intervals based on the sampling distribution.

Fig 1 visualizes the two different scenarios and four random subsamples of two different sample sizes (n = 30 and n = 150), and displays a scatter plot of the most strongly correlated voxel, as well as the distribution of maximum absolute correlation values obtained in the subsamples. Fig 2 summarizes the results of the full simulation. Panel (a) shows the positive relation (by construction) between sample size and the average statistical power, for both the WD and SL scenario. The WD curve rises much more slowly, and statistical power for n = 150 is still less than 9%. In addition, the statistical power to detect *at least one* effect is 100% in both the WD and SL scenario, irrespective of the sample size. Panel (b) demonstrates that the mean absolute estimated effect size (for statistically significant voxels) observed in the samples shows much greater inflation relative to the true effects in the WD scenario compared to the SL scenario, especially when sample sizes are small. Note that in the SL scenario (due to the use of an uncorrected threshold and hence partially due to the effect of false positives) larger sample can actually underestimate the true effect size. In (c), we observe, predictably, that the percentage of significant voxels shows a positive relation with sample size, very comparable to statistical power. For SL samples this reaches a ceiling in line with the true full sample effect, while for the WD subsamples, there is a steady increase in the percentage of significant voxels, yet there is still a large discrepancy (underestimation) of the true full sample effect. Panel (d) demonstrates that the mean overlap (dice coefficient) of subsequent replications shows a positive curve in both scenarios, but rises much more slowly in the WD scenario, again, a near identical pattern to the statistical power results. This thus indicates that there is clear link between statistical power and replicating the spatial pattern of an fMRI study. Note that, because of the uncorrected threshold, and hence the inclusion of false positives, the dice coefficient in the SL scenario does not reach 1.

Overall the results thus show a clear difference in statistical power, effect size estimation and reproducibility for the two different scenarios. The SL scenario performs adequately with common sample sizes of n = 20–30, yet the WD scenario performs poorly on these measures even with much larger sample sizes of n = 100–150. In section B of the S1 Supplementary Analyses we further discuss significance threshold as this has differential effects depending on the particular brain-behavior scenario. For instance, the use of a more stringent threshold (controlling the FDR, q = 0.05; Figure B of S1 Supplementary Analyses) has a positive effect on the replication dice coefficient in the SL scenario, but is detrimental in the WD scenario. Lastly, the two scenarios thus differed on two factors (effect size and distribution), and are merely meant to illustrate a common dichotomy in the thinking about brain-behavior associations. Other scenarios are certainly possible (e.g. a “strong diffuse” or “weak localized” scenario), but we regard them as less likely. The results of the simulations are discussed in relation to the empirical data (see section 3.2 below) in section 4.

### 3.2 Empirical data: Human Connectome Project

The results from the simulations illustrate the heightened impact of low statistical power on conclusions in the WD condition, and the deleterious effects of using smaller sample sizes. Next, we aimed to investigate which of the two scenarios is more comparable to empirical data (See reference [6] for a similar approach). Increasing efforts in large-scale data acquisition and open data sharing have led to the public availability of several very large fMRI datasets [53]. Here we use data from the putative “social cognition” task of the Human Connectome Project (HCP; [54]). We treat the full sample of the “n = 500 release” as an approximation to the true effect, from which samples are drawn and compared to the results to the simulation analysis. While a sample of 500 is indeed very large by fMRI standards, do note that the results presented here are still likely to be biased in the direction of the SL scenario, as the sampling variance of observed effects is undoubtedly still decreasing well past n = 500. Thus, our results are likely to convey an optimistic assessment of reality.

#### Methods

Participants (N = 485) performed a movie-watching task putatively assessing social cognition; detailed information on the task and the preprocessing pipeline can be found in previous publications [54,55]. The scanning protocol was approved by Washington University in the St. Louis' Human Research Protection Office (IRB # 201204036, “Mapping the Human Connectome: Structure, Function, and Heritability"). Briefly, short videos were presented of abstract objects (circles, triangles) that either interacted with one another in biologically plausible ways (Theory of mind or TOM condition), or moved randomly on the screen (Random control condition, CON). The fully preprocessed TOM>CON contrast images of the “500-subject release” were used in the current analyses (we also conducted equivalent analyses of the other HCP functional tasks, with virtually identical results). First, we focused on the experimental (i.e., within-subject) differences between conditions. We generated our best estimate of the true effects by analyzing the full sample (n = 485). We then drew 16 random samples of n = 15 subjects in order to illustrate the difference between the full sample (“the true effects”) and subsample effects. Second, we conducted a between-subjects analysis in which we correlated, at each voxel, the activation estimates for the TOM>CON contrast with a measure of agreeableness, a personality trait associated with cooperative and affiliative behavior, as measured with the NEO-FFI [56]. In this second analysis, 8 random samples were drawn of n = 30, to illustrate the difference in distribution of the full sample (“true”) and subsample effects. For both analyses an uncorrected threshold of *p* < .001 was used.

#### Results

**Fig 3:**
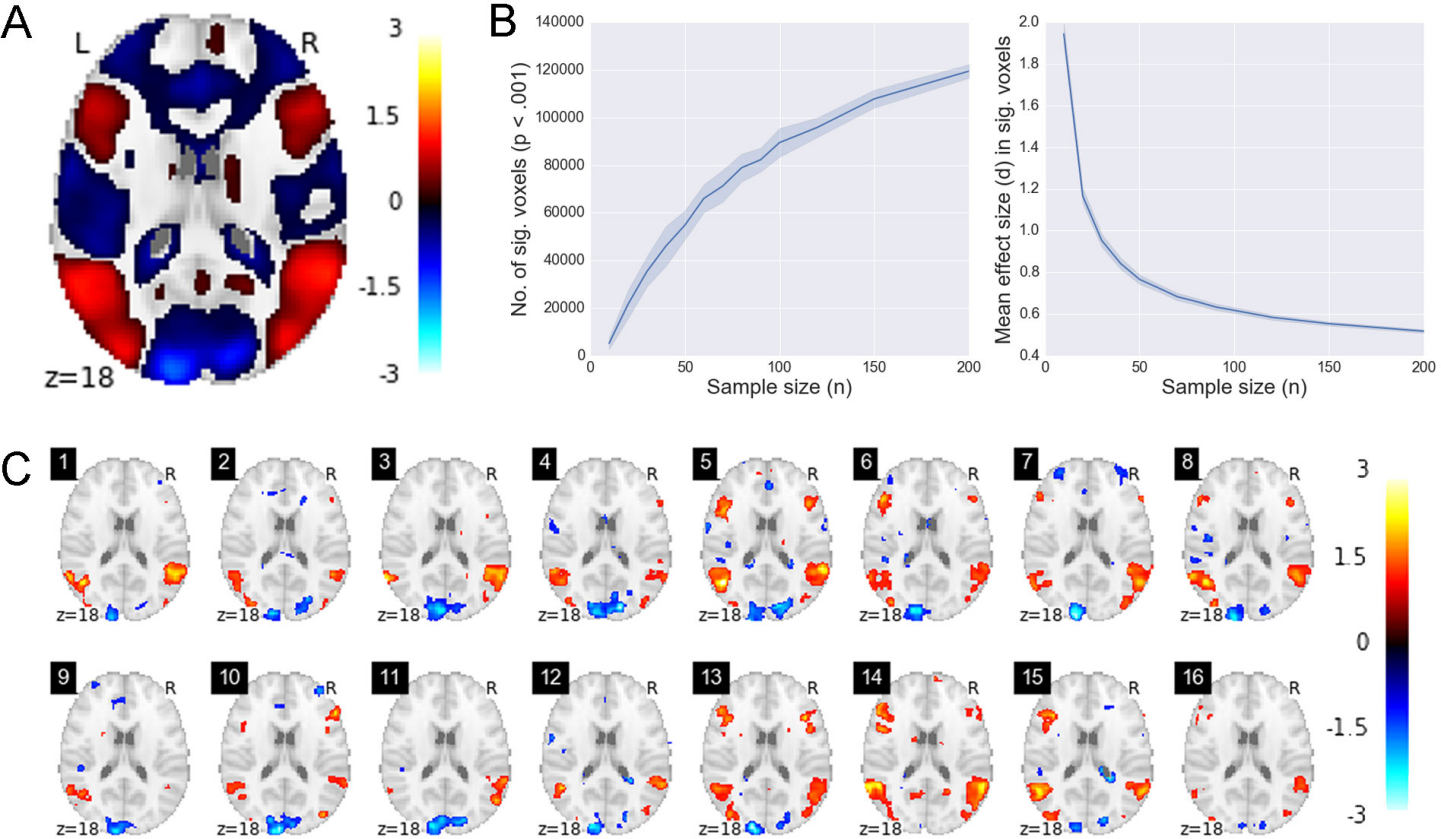
Main effect of the HCP social cognition task. (a) effects for the TOM>CON contrast in the full sample (n = 485). The colors reflect effect size (Cohen's d). (b) number of significant voxels and mean d in significant voxels as a function of subsample size. (c) Results of TOM>CON contrast for 16 random subsamples of n = 15. TOM; theory of Mind. CON; control condition.

**Fig 4:**
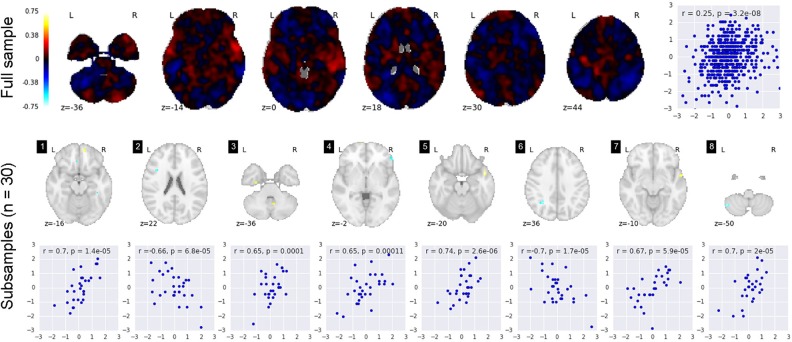
Correlation between the HCP social cognition contrast and the personality trait agreeableness. The top row is the full sample (n = 485), the scatterplot at the right displays the single strongest voxel (r = .25). The bottom row shows 8 random subsamples of n = 30. Each one shows a slice through the brain where effects are "detected" and the scatterplot shows the single most strongly correlated voxel.

Fig 3 shows the results of the HCP social cognition task for the TOM>CON contract. The full sample shows a distributed effect of the experimental manipulation (Fig 3A). The relation between sample size and number of significant voxels shows an obvious positive relation, while sample size and effect size display a negative relation (Fig 3B). Fig 3C illustrates the pattern of activation for samples of n = 15. Subsequently, we investigated the relationship between individual differences in the personality trait Agreeableness and the magnitude of activation in the TOM>CON contrast. Fig 4 illustrates the results. The top row is the full sample (n = 485). The scatterplot at the right displays the single strongest voxel (r = .25). The bottom row shows 8 random subsamples of n = 30. Each one shows a slice through the brain where effects are "detected", and the scatterplot again shows the single most strongly correlated voxel. It is important to note that the correlation analyses used here are sensitive to outliers which can further bias the estimates, especially for small sample sizes [57,58].

The results displayed in Figs 3 and 4 underscore the critical importance of analysis type and research question when considering the likely power of an analysis. The within-subject effects from the TOM>CON contrast (Fig 3) are, in this case, large enough that many key regions can be reliably detected even with samples of 15 subjects. This is likely due in part to the relatively high-powered nature of within-subject tests, and in part to the “strong” nature of the experimental contrast (i.e., the large difference in stimuli in the two conditions). In marked contrast, however, the individual differences analyses (Fig 4) suggest a picture much more consistent with the “weak diffuse” scenario in our simulations. Subsamples of n = 30 virtually never identify the same statistically significant correlations with trait agreeableness, and effect sizes in detected regions show a marked degree of inflation. We discuss the implications of such findings in greater detail in the next section.

## 4. Causes and consequences of low power

Based on the results from the previous section, we discuss four problems associated with conducting underpowered mass-univariate fMRI analyses, including (I) a high rate of missed effects; (II) biased estimation of effects, and particularly, inflation of significant effects; (III) propensity to draw incorrect inferences about underlying neural architecture; and (IV) difficulty arriving at a cross-study consensus about the neural substrates of cognitive-affective functions, but especially between group differences (e.g. patients and controls) or brain-behavior correlations (e.g. personality traits).

### 4.1 High rate of missed effects

As mentioned before, the most obvious problem associated with the use of relatively stringent correction levels is that, by minimizing false positives, or Type I error, one necessarily increases the rate of false negatives, or Type II error. When it comes to the average power to detect true effects, the power in the WD scenario remains below 9% even with 150 subjects (see Fig 2A). The SL scenario shows much higher statistical power (85% for n = 20), and power also increases much more rapidly with sample size. Of course, the power to detect *at least one* effect is typically much higher than this, due to the enormous search space. As is made clear in Figs 1 and 4, even extremely underpowered analyses will generally reveal *some* significant activations when true effects are very weak but also very widely distributed. Identifying only a small fraction of genuine activations—and with highly misleading effect size estimates at that—is, we would argue, not what most researchers are really after. Yet such findings may well be sufficient for a publication (see Box 1)—and, counterintuitively, may even appear to tell a more compelling story than the results produced by a much higher-powered analysis (consider the seeming simplicity of the strong, focal correlations revealed in the subsamples in Fig 4, as compared to the weak, diffuse effects apparent in the full sample). In short, unless one believes that effect sizes are consistently huge in fMRI studies (reflected in the SL scenario), one should accept that most whole-brain fMRI analyses are going to overlook meaningful effects far more often than they successfully detect them. The HCP results, at least for the individual differences analyses, are largely in line with the WD scenario.

### 4.2 Biased effect size estimates

The problem of effect size mis-estimation is illustrated in Fig 2B. The overestimation of the effect size is especially problematic in the WD scenario. That is, with smaller samples, statistically significant effect sizes appear to be much larger than the true effect sizes. The “winner’s curse”, as it is often called, stems directly from the dangerous combination of selection bias (i.e., only statistically significant effects are reported) and low power, and will typically cause underpowered fMRI studies to produce results that reflect sampling error to a much greater extent than genuine signal (for additional discussion, see references [3,6,12,59]. This latter point deserves particular emphasis: contrary to what one might intuitively expect, the use of more stringent multiple comparison correction will result in greater overestimation of statistically significant effect sizes (even as it decreases the Type I error rate). The HCP data (Figs 3B and 4B) illustrate this issue in empirical data, again in line with the WD scenario. For both the within-subject effects (social cognition) and between-subject effects (correlation between social cognition and Agreeableness), small samples considerably overestimate the actual effect size.

### 4.3 Misleading inferences about brain organization

Perhaps the most striking feature of the simulation results (Figs 1 and 2) is that, when one’s sample size is relatively small (e.g., n = 30), whole-brain analyses are almost guaranteed to produce sparse, seemingly large effects irrespective of the true underlying statistics. Put differently, observing highly localized, extremely strong brain-behavior correlations in a small sample tells one almost nothing about the strength and spatial extent of the true effects in the population (cf. reference [3]). This result follows directly from the effect size mis-estimation discussed in the previous section. The analyses of the HCP social cognition data further underscores the point: when randomly subsampling 30 subjects at a time from the full dataset, each sample drawn displays much stronger effects in statistically significant voxels, yet shows many fewer significant voxels (see Fig 4). Thus, when the statistical power is low, the apparent spatial selectivity of one’s results provides little information about the actual distribution of underlying effects. For this reason, most reports of “selective activation” found in the fMRI literature should be approached with skepticism, as most studies making such claims lack sufficient statistical power to distinguish genuinely selective activation from the illusory selectivity induced by the combination of high Type II error and inflated effect sizes. We may refer to this as the *localizationist bias* of typical whole-brain, mass univariate fMRI analyses.

### 4.4 Difficulty achieving consensus

As many other commentators have noted [60–62], few practices are more damaging to scientific consensus-building than a habitual reliance on underpowered studies in which only a fraction of the available results are reported (i.e., only those voxels or regions that attain statistical significance). This point is illustrated by the simulation results presented in Fig 2D, which shows the spatial overlap of significant voxels in subsequent samples, displayed again as a function of sample size. These results are strongly related to statistical power: in other words, underpowered studies will be less likely to replicate [24]. The HCP social cognition and agreeableness results underscore this notion. The small samples drawn from the full sample show different effects each time.

A deleterious consequence of running underpowered studies is thus that researchers will frequently be led down garden paths in futile attempts to replicate “strong” previous findings. Because there is a tendency to take reported effect sizes at face value, researchers who seek to replicate previous effects are likely to overestimate the strength of those effects, leading to excessively optimistic power calculations when conducting follow-up studies. In reality, the follow-up study may have very little power, and the investigator is left having to write a discussion section explaining why the replication attempt was unsuccessful. A standard explanation for such failures is that there must be some critical experimental difference in the replication study that “moderates” the effect—e.g., a difference in the demographic composition of the sample, or the use of a different stimulus modality. This observation often then leads to third, fourth, and fifth studies designed to delineate the boundary conditions under which the original effect holds. Over time, this common pattern will produce the appearance of “mixed findings” in the literature. Rarely is the possibility considered that the original effect may have simply been overestimated, and that the true effect may be too small to consistently replicate without acquiring much larger samples (for a discussion see reference [62] and see [63] for an Bayesian perspective of the reproducibility project in psychology that illustrates the problem empirically). Indeed, consideration of the WD scenario in Fig 1 raises the worrisome prospect that if the underlying effects are very weak, but spatially diffuse, even studies with hundreds of subjects may fail to produce consistent results. This state of affairs may be common in, for example, the personality neuroscience literature, where even very large structural and functional MRI studies have largely failed to identify robust neural correlates of personality traits that are known to be temporally stable and highly heritable (for a review, see reference [64]).

## 5. Discussion: Estimating and increasing power

We have discussed what we regard as the four major problems of underpowered fMRI studies. We suspect that these problems are very likely endemic to much, and perhaps most, fMRI research. That is, they are not the sole province of a minority of careless fMRI researchers who employ sloppy practices, and are instead liable to affect most mass-univariate analyses of fMRI data. Achieving adequate power is likely to require a multi-faceted strategy that relies on a number of different approaches. In this section, we discuss five (non-exhaustive) classes of procedures that can help identify and ameliorate the negative consequences of low statistical power in fMRI studies.

### 5.1. Power analyses and confidence intervals

Although power analysis is considerably more complicated in fMRI studies than in behavioral research, a number of sophisticated software packages have been developed in recent years [5,10,65]. The novel neuropowertools.org for instance allows users to import maps uploaded to the public NeuroVault.org repository [5]. Alternatively, researchers can simply use any standard power analysis program (e.g. reference [66]) as long as care is taken to use realistic parameters, rather than optimistically assuming a best-case scenario. For calibration purposes, it may be instructive to consider that the power to detect a medium effect of r = 0.3 in a small sample (n = 30) at a relatively liberal threshold (*p* < .001; the discussed common practice in fMRI) is only 4.1%!

Assuming data have already been acquired, there are a few basic procedures that can help limit the impact of the mis-estimation issues discussed in section 4.2. *Reporting standardized effects and confidence intervals* can help gauge the importance and/or plausibility of a given effect. P-values alone rarely provide an adequate sense of the uncertainty surrounding any effect size estimate, and confidence intervals are superior to classical hypothesis tests in nearly all respects [21,62,67,68]. For instance, a ‘huge’ correlation of r = 0.88, *p* < .001 seems much less impressive given knowledge that the 99.9% CIs around that point estimate happens to range from 0.07–0.97. In addition, a method to provide post-hoc power estimations based on the whole-brain distribution of observed effects has been proposed [4].

### 5.2 Increase sample size

Of the three variables that essentially determine statistical power, sample size and significance threshold are by far the most “controllable” by researchers. The most obvious way of increasing statistical power therefore is to increase sample size. Although it may be difficult (for instance when studying patient populations) and costly (an hour of fMRI data acquisition typically costs anywhere between $300 and $1,000) to acquire large amount of subjects, one should try to maximize these efforts. For instance, when deciding about a new study protocol our sense is that the decision is often made to include more tasks per subject (in order to maximize the number of potential research papers). However, for the reason outlined in the previous sections, we would argue that trying to maximize the number of subjects per scan time unit (two subjects per hour, three in two hours etc) should be given equal consideration [10]. Similar cost considerations may also lead some researchers to conclude that they would be better off using very large convenience datasets like the HCP rather than acquiring new datasets that are carefully tailored to the question at hand but may be hopelessly underpowered.

### 5.3 Adjust the significance threshold

By definition, another way is to increase power is to use a less stringent significance threshold. Current procedures for multiple comparisons correction focus primarily on controlling the Type I error or false positive rate. As a consequence, Type II error rates are often unacceptably high. To strike a better balance between Type I and Type II errors, some researchers have advocated using conventional uncorrected thresholds such as *p* < .001 or *p* < .005 [41]. However, such an approach is unprincipled, in that it fails to take into account the specific parameters of each analysis (such as smoothness of the data; [42] which can have large consequence for the false positive rate and statistical power. An uncorrected threshold is also susceptible to unintentional p-hacking [69,70] since researchers can easily choose between several different thresholds. Moreover, while uncorrected thresholds may maximize the power to detect *at least one* effect (see Fig 2A), under typical parameter regimes, they are still extremely underpowered to detect most of the effects, and are thus susceptible to the *localizationist bias* discussed above. In the section C of the S1 Supplementary Analyses we discuss the possibility of balancing the error rates (i.e., the ratio between type II/type I error) in a more principled way. These results show that in order to *truly* balance the error ratio to, for instance, an acceptable ratio of 4 (e.g. achieved by the typical 20% type II error and 5% type I error rate) in a study of n = 30 drawn from a “Weak Diffuse” effect size scenario one would have to adjust the significance threshold to *p* < .34 uncorrected!

### 5.4 Reduce the number of statistical tests

#### Hypothesis-driven tests

A widely advocated strategy for increasing the power of fMRI analyses is to rely on hypothesis-driven analyses that focus on specific “regions-of-interest” [71]). When used in a principled manner, hypothesis-driven analyses can substantially ameliorate the problems associated with having to correct for multiple comparisons, and we encourage researchers to use ROI based analyses when appropriate. However, the use of ROI analyses is not a license to ignore the need to correct for multiple comparisons. One cannot test a dozen ROIs and interpret statistically significant results as though the false positive rate were still only 5%. Moreover, it is rarely possible to determine from published reports exactly how and at what stage ROIs were selected for analysis, or whether the reported ROIs represent a comprehensive list of those that were tested [6]. Pre-registration of analyses and study plans, which is common for clinical trials [72] could potentially help, but has not yet been widely adopted in cognitive neuroscience (but see reference [73]). Lastly, it is important to realize that ROI analyses alone cannot quantify the specificity of any identified effects—e.g., the interpretation of a statistically significant effect in an amygdala ROI would likely change if one knew that 60% of the rest of the brain also shows the same effect (see section D of the S1 Supplementary Analyses for a straightforward potential solution to this problem; the selectivity index).

#### Multivariate and model-based fMRI analyses

If one adopts a more distributed neural implementation view (in line with the weak distributed effect scenario), there are a host of methods that can help increase statistical power simply by not having to correct for multiple comparisons to the same degree [74]. For such methods to ameliorate the statistical power problem however, all variables of interest (brain data) need to be considered in *one* model. A review of these methods is well beyond the scope of this paper, and we simply briefly mention a few options and examples. Model-based approaches can be used in a univariate way (e.g. predict working memory performance [75]) or multivariate analyses (e.g. to predict pain [76] and negative affect [77]. These later *machine learning analyses* aim to infer for instance mental states or psychiatric disorder diagnosis (e.g. references [78,79]) from observed patterns of brain activity. Another class of methods concerns latent variable and *data reduction approaches* such as independent components analysis (ICA; e.g. [80,81]) or structural equation modeling (SEM; [82]) Such approaches typically increase power by reducing the number of variables tested (e.g., 40 ICA components rather than 200,000 voxels), affording the use of more liberal statistical thresholds. Canonical Correlation Analyses (CCA) is another powerful multivariate tool to address the correspondence of two datasets, see for instance refs [83,84]. *Graph or network theory* forms another informative and elegant alternative to *mass* univariate analyses [85–88]. Here the focus is on properties of connectivity networks rather than localized activity. When this approach is for instance used to “summarize” brain functioning to high-level features such as efficiency or modularity, the multiple comparison problem is largely avoided, and consequently its associated decrease in statistical power.

### 5.5 Meta-analysis, mega-analysis, and synthesis-oriented approaches

To varying extents, the practices suggested above can all help to at least identify and sometimes ameliorate the effects of low power in individual studies. However, since sample sizes in fMRI studies are only increasing moderately [6], power in individual studies is likely to remain inadequate in many cases. Given this fundamental constraint, we suggest that the best hope for accurate and comprehensive reporting of neuroimaging results lies in large-scale efforts to integrate and formally synthesize the published research literature—i.e., to adopt a cumulative approach to the study of human brain function [89]. *Meta-analysis* has been used to map the neural correlates of numerous tasks and cognitive functions, including everything from word naming to working memory performance [90] to different emotional categories [91,92]. Critically, such studies can compensate for the limited power of individual neuroimaging studies by identifying brain regions that show consistent activation across many hundreds of studies, even when base rates of activation in any given region are relatively low. In recent work, we have developed an automated framework for meta-analysis (Neurosynth; http://neurosynth.org; [93]) that uses simple text-mining algorithms to automatically generate hundreds of large-scale meta-analyses of the fMRI literature. These maps converge reasonably well with manually produced meta-analyses [93,94] and this meta-analytic data has been incorporated to the analyses of single studies (e.g. [95,96]). Other large-scale collaborative efforts include *mega-analyses* (such as the ENIGMA consortium [97]) and very promising are the various initiatives for open-data sharing [53] such as HCP used in the current paper and the Functional Connectomes Project (FCP/International Neuroimaging Data sharing Initiative (INDI) [98] and the UK Biobank [99].

## 6. Conclusion

We have argued that low statistical power presents a serious—and despite the increased attention given to the topic, underappreciated—threat to continued progress in the field of neuroimaging. This is particularly problematic when conducting massively univariate whole-brain analyses, when dealing with between-subject effects, and when effect sizes show weak but spatially distributed effects. Our analysis of one of the largest currently available public fMRI datasets demonstrates that, in principle, a “diffuse and very weak” model of brain-behavior correlations is entirely compatible with the seemingly large, and highly localized effects routinely produced by very small fMRI studies. In such a regime, failure to ensure adequate power is likely to result in a failure to detect the vast majority of meaningful effects, to produce gross inflation of statistically significant effect sizes, and to impede the rate at which neuroimaging researchers can build consensus across multiple studies. Unfortunately, because of the high cost of data collection, increasing power in neuroimaging studies is not a trivial exercise. We have suggested a number of practices researchers could implement in individual studies in order to either directly increase statistical power, or to ameliorate the negative consequences of low power by minimizing misinterpretation of results from underpowered analyses.

## Supporting information

S1 Video Sampling from a medium effect size. In order to illustrate the effect of sampling error and the rate at which effect size estimation stabilizes several subsamples are drawn from a very large sample (n = 10,000). The upper panel of the video shows a correlation of r = 0.3 between two variables in the full sample. From this full sample, 30 subsequent subsamples are drawn, with a range of different samples sizes (n = 10–150). Every time a sample is drawn, it is shown on the upper panel, and the correlation coefficient is plotted on the lower panel. If the correlation is statistically significant (*p* < .05) the correlation is displayed in orange, if not it is shown in blue. The video illustrates that (I) for smaller sample sizes the proportion of significant correlations (compared to the total of 30 correlations) is much smaller, obviously reflective of lower statistical power (II) significant correlation coefficients of small subsamples are much higher than the correlation coefficient in the full sample, while for larger subsamples these two become more comparable (III) the range of correlation coefficients is much larger for smaller than larger subsamples, corresponding to the confidence interval.
(MOV)

S1 Supplementary Analyses (PDF)

S1 Data Data of the results presented in Fig 2. (ZIP)
