# Supplementary material for: The relation between statistical power and inference in fMRI
Source: PLoS One. 2017 Nov 20;12(11):e0184923. doi: 10.1371/journal.pone.0184923 (PMC5695788; doi:10.1371/journal.pone.0184923)
Supplement: S1 Supplementary Analyses — (PDF) [file pone.0184923.s002.pdf]

The relation between statistical power and inference in fMRI

Henk R. Cremers, Tor D. Wager, Tal Yarkoni

S2 Supplementary Analyses.

A. Average and *at least one* effect statistical power
B. Simulation results using a FDR correction
C. Error Rate
D. Selectivity Index

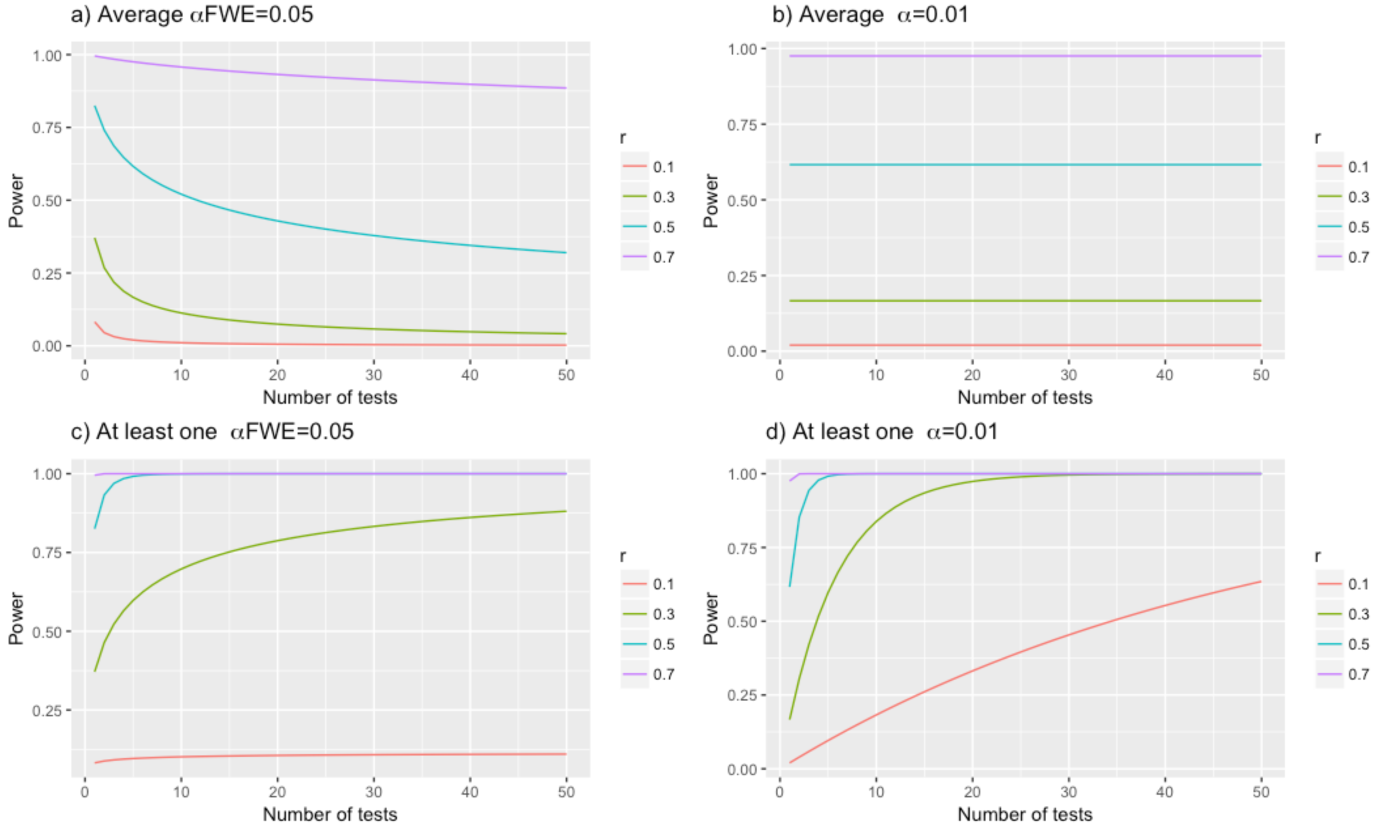

Fig A. Average and *at least one* statistical power for different effect sizes and number of tests.

This figure illustrates the relation between statistical power and the number of tests (1-50) for several standard effect sizes (Correlation coefficients; Pearson's r, range 0.1-0.7) and for corrected (αFWE = 0.05, Family Wise Error; FWE, estimated by applying a Bonferroni correction) and uncorrected (α = 0.01) significance thresholds, for a sample size of n = 30. **a)** The average power for αFWE = 0.05 **b)** The average power for α = 0.01 **c)** The power to detect at least one effect for αFWE = 0.05 **d)** The power to detect at least one effect for α = 0.01.

B. Simulation results using a FDR threshold of q = 0.05.

Figure B shows the results of the subsampling analyses (3.1) when a false discovery rate [1] threshold of q = 0.05 is applied. The results naturally show for instance that the statistical power is lower with this more stringent threshold compared to the uncorrected threshold of $p < .01$, in both scenario's. However, when considering the effect size estimation and dice coefficient, there is a clear differentiation in effects for the two scenarios compared to applying an uncorrected threshold. When applying the FDR correction (compared to the uncorrected threshold of $p < .01$) the effect size estimation becomes much closer to the full sample's range in the SL scenario, while in the WD scenario it leads to stronger overestimation of the full sample effect sizes. Similarly, the dice coefficient becomes higher in the SL scenario but lower in the WD scenario. The appropriate choice of a significance threshold thus clearly depends on the expected effect size and effect size distribution, see also section C.

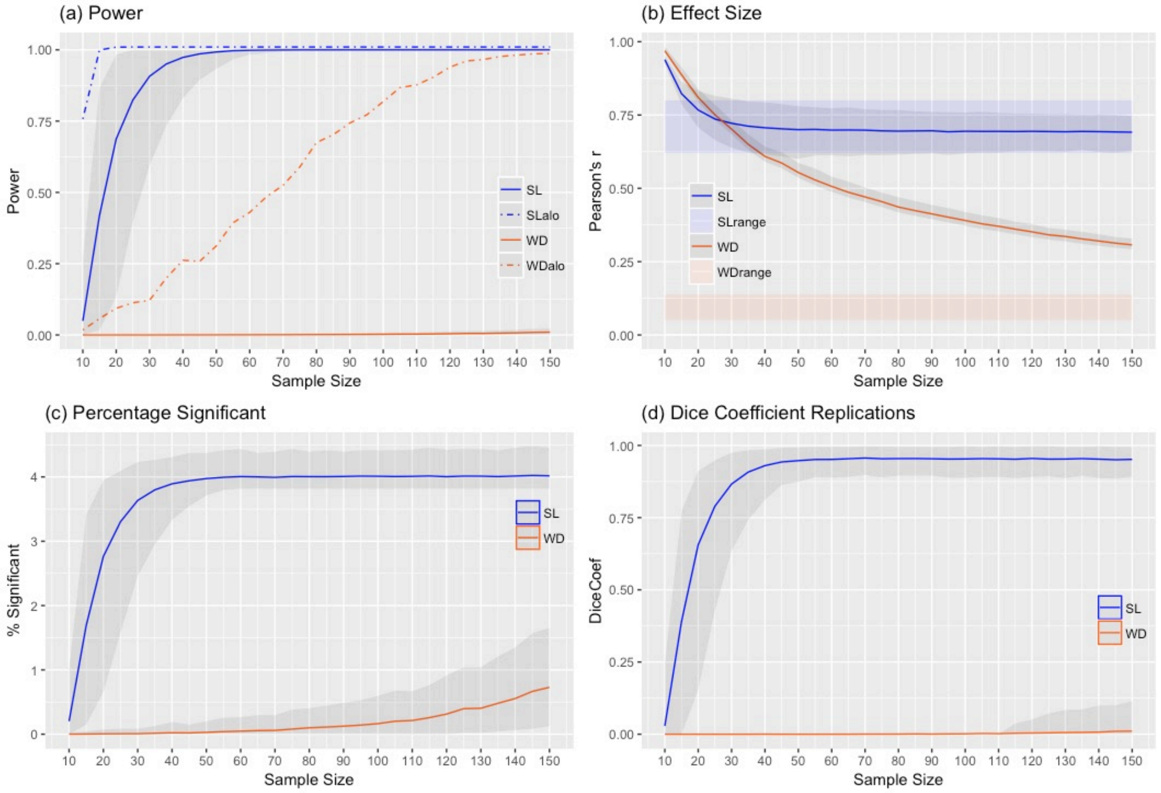

Figure B. Results of the simulations using a FDR threshold of q=0.05

Relation between sample size (n) and **(a)** *average* (solid line) and *at least one* (alo; dashed line) statistical power; **(b)** detected effect size (Pearson's r) in the samples, and the full population effect size range (shown as a transparent color bar); **(c)** Percentage of voxels below the threshold; **(d)** mean dice coefficient: overlap of significant (q = 0.05) voxels between two subsequent replications. SL; Strong Localized effects, WD; Weak Diffuse effects. The shaded grey area around the estimates reflects the 95% confidence intervals based on the sampling distribution.

C. Significance threshold and the Error Ratio

Typically, when determining what statistical threshold to use, researchers give consideration either exclusively to the Type I error rate (eg. FWE), or to the ratio between the Type I error rate and the true positive rate (like the mentioned false discovery rate). Although both approaches have their strengths, neither gives any direct consideration to the Type II error rate, effectively implying that false negatives are of little or no concern. One way to illustrate the balance between these two error rates is the receiver operator characteristic (ROC), see fig C(a) which shows the sensitivity (power, 1-Type II error rate) as a function of the 1-specificty (Type I error rate) for r = 0.1 (rounded average of the weak diffuse scenario) and r = 0.7 (rounded average of the strong localized scenario) with n = 30. Yet another way to illustrate the problems associated with stringent correction for multiple comparisons is to quantify the Type II to Type I error ratio (ER). For example, given a threshold of $p < .001$, for instance, the error ratio for a correlation coefficient with n = 30 remains close to 1,000:1 for correlations up to r = 0.3. In other words, for every incorrectly rejected null hypothesis, a researcher will miss out on approximately 1,000 true effects. Compare this situation to the "gold standard" 4:1 (ie. 80% power, 5% false positives). One could thus perform a "compromise power" analysis [2] aiming to balance these two types of error. For fMRI such an approach can be extended by using a simple mixture model under which some proportion of voxels *p(ACT)* show "real" (i.e., non-zero) effects and the complement show a null effect and for simplicity we assume that all voxels in set *p* have an identical effect size (the point goes through essentially unchanged given a distribution of effect sizes). Then the ER is simply *p(ACT)* β / (1- *p(ACT)*)α, where β is the probability of a false negative at a given truly-activated voxel (Type 2 error rate, or 1-power), and α is the nominal Type I error rate. Figure C(b) displays the log10 ER as a function also of the 1-Specificty (Type I error rate) for the two scenarios we considered (Weak Diffuse: p(ACT) = 0.7, *r* = 0.1, Strong Localized: p(ACT) = 0.04, *r* = 0.7) for n = 30. The vertical lines indicate at which significance threshold for each scenario the error ratio approximates 4. One can observe that for the WD scenario, the combination of distributed and small effects and low power leads to extremely high error ratio and only at α = 0.34 will the ER drop to 4, while for the SL scenario this is at α = 0.001.

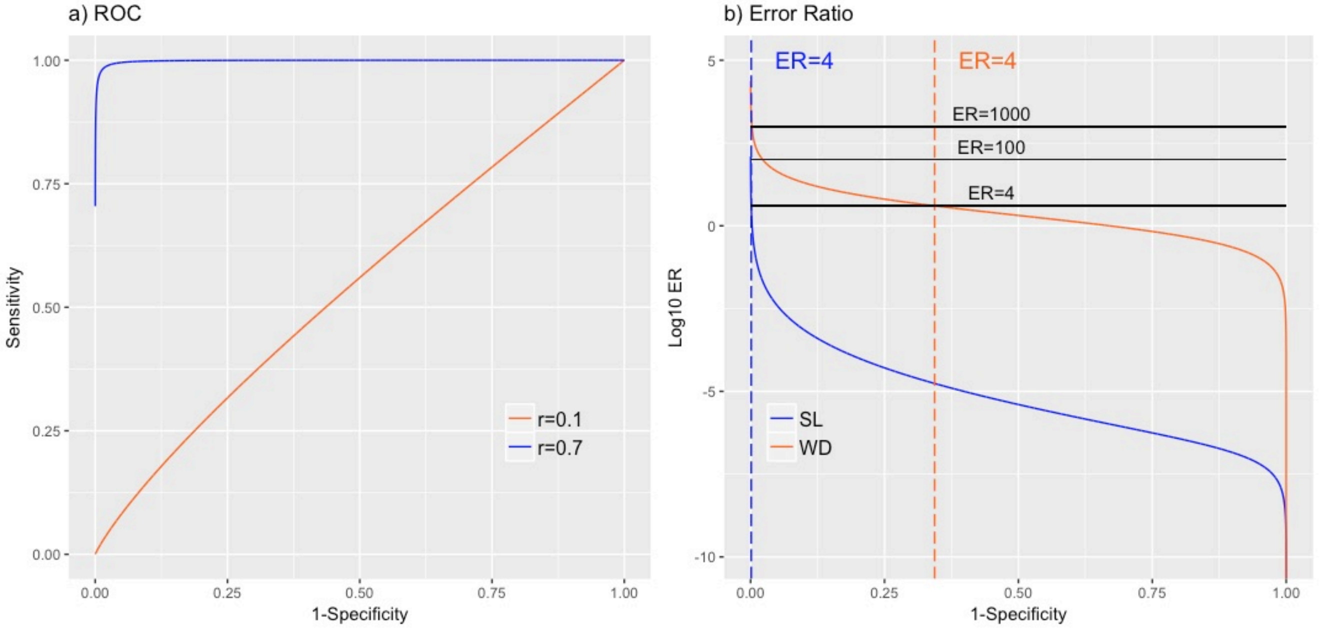

Figure C. (a) Receiver Operator Characteristic (ROC) for two effect sizes (r = 0.1 and r = 0.7) with n=30 and (b) Log10 Error Ratio for the two scenarios both with n = 30. SL: strong localized, WD: weak diffuse.

D. Selectivity Index

The selectively index (SI) is simply the proportion of all other voxels that are statistically significant when tested at the same level as any a priori ROIs. For example, in a study that used a $p < .05$ threshold to test for ROI-level effects, the SI would be the proportion of all other voxels anywhere in the brain that were activated at $p < .05$. This quantity has the benefit of being easy to calculate and report, and would provide a much needed baseline for evaluating specificity claims. For example, a finding of statistically significant activation in an a priori amygdala ROI would be interpreted very differently depending on whether 3% or 30% of other voxels showed the same effect when tested at the same threshold.

References

1. Benjamin Y, Hochberg Y. Controlling the false discovery rate: a practical and powerful approach to multiple testing. J Royal Statistical Society; 1995.
2. Faul F, Erdfelder E, Lang AG, Buchner A. G* Power 3: A flexible statistical power analysis program for the social, behavioral, and biomedical sciences. Behav Res. Springer; 2007;39: 175–191.
